# Stewart–Treves syndrome: a case report

**DOI:** 10.11604/pamj.2014.19.2.4178

**Published:** 2014-09-01

**Authors:** Anis Benmansour, Saad Laanaz, Abdeslam Bougtab

**Affiliations:** 1 Department of Surgical Oncology, National Institute of Oncology, Rabat, Morocco; 2 Department of Medical Oncology, National Institute of Oncology, Rabat, Morocco

**Keywords:** Stewart–Treves, syndrome, angiosarcoma

## Abstract

The Stewart-Treves syndrome was first described in 1948, it's an angiosarcoma developed on a longstanding lymphadenomatous limb, more often after radical mastectomy. Diagnosis is made on skin biopsy and the prognosis is poor when radical surgery can't be performed. We report the case on a Stewart-Treves syndrome in a sixty-six years old woman who underwent radical mastectomy for breast carcinoma ten years earlier. Surgery was not feasible at the time of diagnosis, and we lost touch of the patient even if chemotherapy was decided. Radical surgery is the best treatment to date for this rare disease. Conservative surgery with adjuvant radiotherapy is also possible. Systemic chemotherapy is reserved for locally advanced unresectable and metastatic forms. We advocate long term follow-up for every post mastectomy lymphedema to diagnosis this fatal disease when curable.

## Introduction

Stewart-Treves syndrome is an angiosarcoma developed from longstanding lymphedema due to axillary lymph node dissection following a radical mastectomy. It was first reported by Stewart and Treves in 1948 [1]. In their study, they reported a series of six patients who had developed angiosarcoma in their lymphedematous extremities after radical mastectomy. The controversy persisted for the exact mechanism of Stewart-Treves syndrome; some authors have favored the hypothesis of epithelial skin metastases of breast cancer Treaty, as amended by stromal edema environment [2, 3]. But the presence of a vascular proliferation tumor associated with the appearance of antifactor VIII positive sign the original angiosarcomatosis. In addition, the Stewart-Treves syndrome has not only been described in post mastectomy, it can be post radiotherapy, congenital, post trauma or post burn.

## Patient and observation

**Figure 1:**
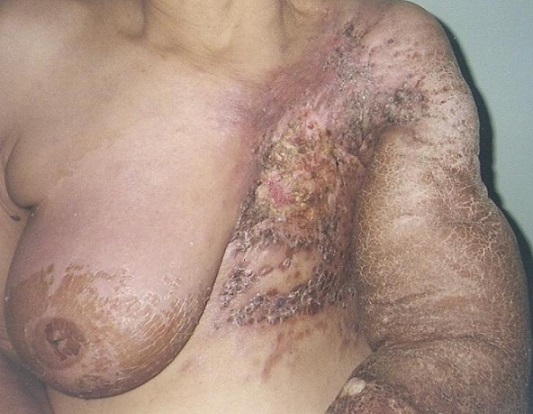
Angiosarcoma extending on the chest wall

**Figure 2:**
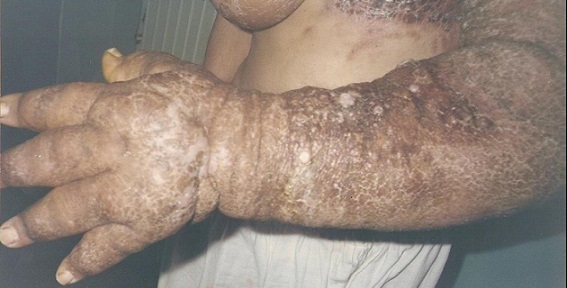
Stewart-Treves syndrome of the left arm

We report the case of a sixty-six years old woman who underwent radical mastectomy for breast carcinoma ten years earlier. She was complaining of chronic lymphedema of the left arm five years ago and developed on this lymphoedema, cutaneous lesions (Figure 1, Figure 2) that extended to the chest wall and was not accessible to a radical surgery. Skin biopsy confirmed the diagnosis of angiosarcoma. No metastasis were found elsewhere. A treatment by chemotherapy was decided but we lost touch of the patient.

## Discussion

The Stewart-Treves syndrome was first described by Fred Stewart and Norman Treves in 1948, they reported six cases of angiosarcoma that arose from areas of lymphedema as a consequence of mastectomy. Today, there are over 400 cases of Stewart-Treves reported in the literature. The prevalence is estimated at approximately 0.45% in patients who survive longer than five years after a mastectomy [4]. The incidence has been decreasing recently, most likely due to changes in operative therapy and radiotherapy in breast cancer. Conservative surgery to minimize loss of breast tissue, coupled with less axillary radiation treatment, have led to a reduction in chronic lymphedema to as low as 4%. This decreasing has also been aided by the advent of sentinel lymph node biopsy.

The Stewart-Treves syndrome is characterized clinically by the occurrence, at a lymphadenomatous member of tumor infiltrating lesions, nodules, often multiple and purple, painless, rarely ulcerated. The series of Woodward et al. [5], recording 162 cases of lymphangiosarcomas after mastectomy, helped to better define their natural history. The onset is usually late, an average of 10 years and 3 months after mastectomy, with extremes ranging from 1 to 26 years. The involvement of adjuvant breast irradiation remains unclear in the pathogenesis of Stewart-Treves syndrome after mastectomy. However, the practice of axillary irradiation after an axillary lymph node dissection is well-known to increase the risk of post-treatment lymphoedema. Thereby reducing the incidence of Stewart-Treves syndrome involves to better define the indication of adjuvant radiotherapy for breast cancer. Furthermore, Woodward et al. [5] noted the frequent association (9-19%) to a third cancer, most frequently gynecological. In the absence of any specific treatment, the prognosis of Stewart-Treves syndrome remains dark, with a median survival of a few months. Its spontaneous evolution is characterized by major tumor aggressiveness responsible of locoregional extension by contiguity to the limb and chest wall, associated with metastatic extension represented by the lungs.

The underlying pathophysiology of this disease is still debated. It is postulated that lymphatic blockage encourages the growth of lymphatic vessels through growth factors and cytokines. This is demonstrated by the fact that there is lymphatic vessel proliferation in unaffected areas of the limb [4]. Histological examination with an optical microscope [6] commonly found within a stromal edema, a nodular tumor proliferation, consisting blanks pace vascular cavities, consisting of many carriers endothelial cell atypia. The vascular and conjunctiva nature of these tumors is confirmed by immunohistochemistry, with positive staining for laminin, antibodies against factor VIII, type IV collagen and CD31[7]. Negativity of epithelial differentiation markers (EMA, cytokeratin) finally eliminates the main differential diagnosis, consisting of cutaneous metastases of breast cancer. High level gene amplification of MYC is also a distinguishing feature of cutaneous secondary angiosarcomas, which develops after chronic lymphoedema or irradiation. This is in contrast to primary angiosarcomas, which tend to be present deep in soft tissue and do not have high MYC gene amplification [8].

The scarcity of Stewart-Treves syndrome explains the absence of therapeutic strategy. Treatment of this syndrome is difficult as angiosarcomas are all high grades, and typically metastasise to the lungs and chest wall. In localized forms, the respective roles of surgery, radical or not, local radiotherapy or a combination of both are not precisely determined. The choice of treatment will depend on prognosis of the breast tumor underlying this disease and wishes of the patient. Patients who are treated with amputation rather than radiotherapy have a better prognosis. Despite of this, their overall survival remains very poor with a survival range of 5-8 months [9]. Systemic chemotherapy is reserved for locally advanced unresectable and metastatic forms. Although its therapeutic benefit is little studied, few cytotoxic drugs have indeed been evaluated, including anthracyclines and ifosfamide who are the major drugs in soft tissue sarcoma. Two Japanese teams [10] have reported result of active immunotherapy with recombinant interleukin 2 (IL2). The case is a 76 years old woman who developed angiosarcoma on chronic lymphoedema 11 years after mastectomy treated by local and systemic injections of recombinant IL2, the patient lived 16 months. However, she had received a local parallel irradiation.

## Conclusion

The stewart-Treves syndrome is an angiosarcoma developed on a lymphadenomatous limb, most frequently after radical mastectomy and axillary dissection. The prognosis is very poor, but one should not be nihilistic. We advocate continuous follow-up for every post mastectomy lymphedema with skin biopsy of every suspicious lesion, so radical surgery can be feasible at the time of diagnosis.
